# Supplementary figures and images for: Identification of Prognosis Associated microRNAs in HNSCC Subtypes Based on TCGA Dataset
Source: Medicina (Kaunas). 2020 Oct 13;56(10):535. doi: 10.3390/medicina56100535 (PMC7650743; doi:10.3390/medicina56100535)

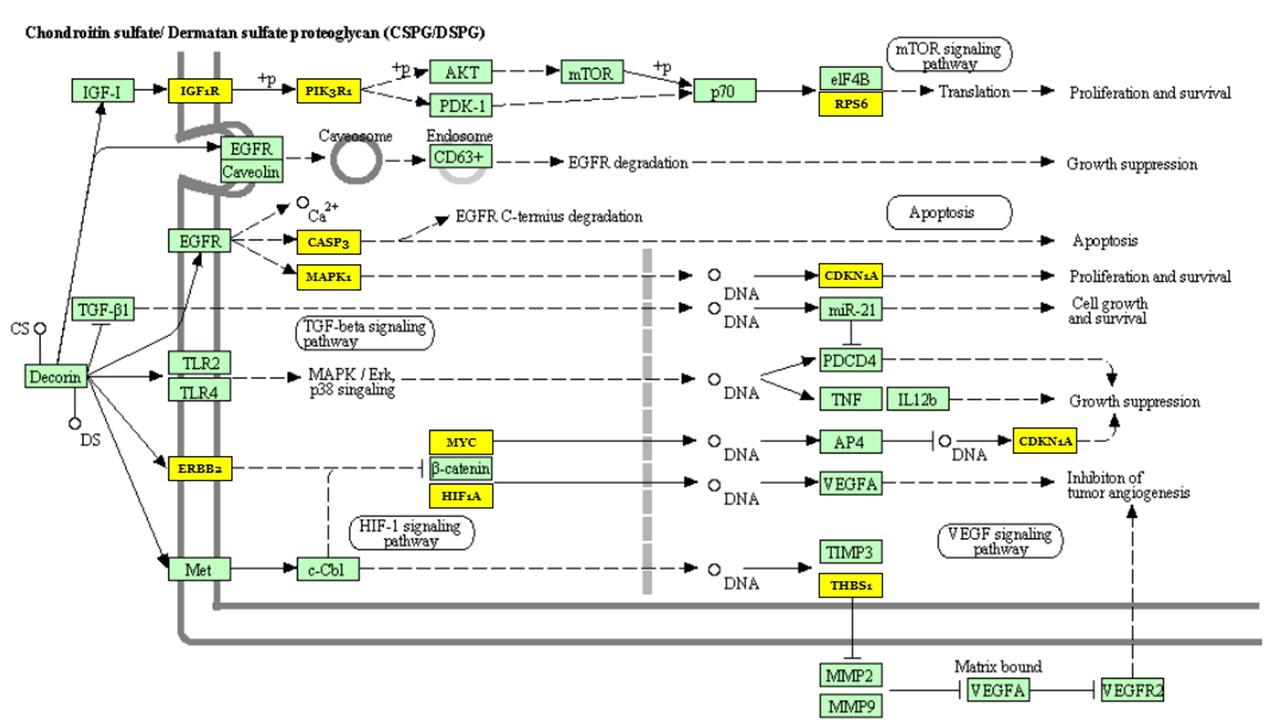

Supplement: Supplementary file 1 [file medicina-56-00535-s001.jpg]
